# Healthcare Resource Utilization for Recurrent *Clostridium difficile* Infection in a Large University Hospital in Houston, Texas

**DOI:** 10.1371/journal.pone.0102848

**Published:** 2014-07-24

**Authors:** Samuel L. Aitken, Tiby B. Joseph, Dhara N. Shah, Todd M. Lasco, Hannah R. Palmer, Herbert L. DuPont, Yang Xie, Kevin W. Garey

**Affiliations:** 1 University of Houston College of Pharmacy, Houston, Texas, United States of America; 2 Baylor St. Luke’s Medical Center, Houston, Texas, United States of America; 3 University of Texas School of Public Health, Houston, Texas, United States of America; 4 Baylor College of Medicine, Houston Texas, United States of America; 5 Merck & Co., Whitehouse Station, New Jersey, United States of America; Cornell University, United States of America

## Abstract

**Background:**

There are limited data examining healthcare resource utilization in patients with recurrent *Clostridium difficile* infection (CDI).

**Methods:**

Patients with CDI at a tertiary-care hospital in Houston, TX, were prospectively enrolled into an observational cohort study. Recurrence was assessed *via* follow-up phone calls. Patients with one or more recurrence were included in this study. The location at which healthcare was obtained by patients with recurrent CDI was identified along with hospital length of stay. CDI-attributable readmissions, defined as a positive toxin test within 48 hours of admission and a primary CDI diagnosis, were also assessed.

**Results:**

372 primary cases of CDI were identified of whom 64 (17.2%) experienced at least one CDI recurrence. Twelve of 64 patients experienced 18 further episodes of CDI recurrence. Of these 64 patients, 33 (50.8%) patients with recurrent CDI were readmitted of which 6 (18.2%) required ICU care, 29 (45.3%) had outpatient care only, and 2 (3.1%) had an ED visit. Nineteen (55.9%) readmissions were defined as CDI-attributable. For patients with CDI-attributable readmission, the average length of stay was 6±6 days.

**Conclusion:**

Recurrent CDI leads to significant healthcare resource utilization. Methods of reducing the burden of recurrent CDI should be further studied.

## Background

*Clostridium difficile* infection (CDI) is the leading cause of hospital-acquired infectious diarrhea in the United States [1]. The healthcare burden of CDI has more than tripled since 1993, with CDI now listed as a diagnosis in over 3% of all U.S. hospital admissions [2]. Additionally, disease recurrence occurs in roughly 20% of initial cases further increasing healthcare resource utilization [3].

Despite the frequency of CDI recurrence, there are limited data examining the healthcare resource utilization in patients with CDI recurrence. Prior studies conducted at large health maintenance organizations demonstrated that 22% of inpatients diagnosed with CDI will have a subsequent re-hospitalization within 6 months with CDI listed as a primary or secondary diagnosis with an attributable cost estimated at $7,179 [4], [5]. However, neither of these studies accounted for readmissions strictly due to CDI recurrence. The purpose of this study was to assess patients with recurrent CDI in order to (1) examine the primary location for which patients with recurrent CDI obtain healthcare and (2) provide estimates for re-hospitalizations attributable to recurrent CDI.

## Methods

This was a prospective, observational cohort study of all consenting adult inpatients greater than or equal to 18 years old between 2007 and 2012 with confirmed CDI at a large (>500-bed) tertiary-care, university-affiliated medical center in Houston, Texas. Patients with CDI were defined as those who had developed diarrhea (defined as ≥3 loose stools in a 24-hour period) [6] and had a positive stool *C. difficile* toxin assay. Prior to 2009, *C. difficile* toxin was identified using an *in vitro* cell-rounding cytotoxicity assay using fresh stool samples [7], while a toxin B polymerase chain reaction (PCR) assay (BD GeneOhm Cdiff PCR; Becton, Dickinson and Company; Franklin Lakes, NJ, USA) was used from 2010 on.

### Ethics Statement

This study was approved by the institutional review boards of Baylor St. Luke’s Medical Center, the University of Texas Health Science Center in Houston, and the University of Houston. All participating patients or their healthcare proxy provided signed informed consent.

### Identification, Treatment, and Follow-up of CDI

Patients eligible for entry into the cohort were identified by a daily review of *C. difficile* stool toxin tests in the clinical microbiology laboratory. Treatment of CDI was performed according to the discretion of the primary treatment team prior to May 2010. Following this date, all patients with severe CDI as defined by a modified version of the severity score proposed by Zar, et al. were placed on oral vancomycin per hospital policy as previously described [8], [9]. Patients with third or later occurrences of CDI were exempt from this policy. No patients in this study were treated with fidaxomicin.

In order to assess for recurrent CDI, patients were followed daily while hospitalized and contacted *via* telephone on a weekly basis for 3 months following discharge from the hospital. Recurrent CDI was defined as new diarrhea with a confirmatory stool toxin test following initial resolution of the prior episode for at least 24 hours and discontinuation of any CDI antibiotics. The reappearance of symptoms while receiving CDI antibiotics was considered a treatment failure and not recurrence. For recurrences identified on a strictly outpatient basis, patients were asked if they had been experiencing diarrhea for and had been told by their treating physician that they have recurrent CDI on the basis of a positive stool toxin test.

### Healthcare Resource Utilization during Recurrent CDI

All patients with an episode of recurrent CDI were assessed to determine healthcare resource utilization. The location at which care was obtained for recurrent CDI was determined and placed into one of four categories: (1) inpatient admission to a general ward, (2) inpatient admission directly to an intensive care unit (ICU), (3) emergency department visit without a subsequent hospitalization, and (4) outpatient care without hospitalization.

For all patients who were hospitalized at the primary study site, general hospital length of stay data as well as ICU-specific length of stay were determined. A CDI-attributable readmission was defined as a readmission with CDI as the primary listed diagnosis in the hospital billing record as well as the presence of a positive stool toxin assay within three calendar days of admission in accordance with the Society for Healthcare Epidemiology of America (SHEA) CDI surveillance definitions [10]. Patients who were readmitted with CDI were additionally assessed for the level of medical care required following discharge. A higher level of care post-discharge was defined as more intensive medical services required in comparison to those prior to the hospital admission (for example, a patient admitted from home discharged to a nursing home).

### Statistical Analysis

All data gathered were stored in a relational database (Microsoft Access, Microsoft Corporation, Redmond, WA). Data were analyzed using descriptive statistics and presented in tabular and graphic form as appropriate. Statistical analyses were performed using Stata v13.1 (StataCorp LP, College Station, TX, USA).

## Results

A total of 372 patients with primary CDI were identified between February 2007 and July 2012. There were no significant differences in the recurrence rates before and after implementation of PCR-based testing for CDI (P = 0.97 by χ^2^ test). The mean age of the overall cohort was 62.3±17.7 years and 54.6% (95% CI: 49.6%–59.5%) were female. Sixty-four patients (17.2%, 95% CI: 13.4%–21.0%) experienced one or more episodes of recurrent CDI. Of these 64 patients with recurrent CDI, 12 (18.8%, 95% CI: 9.2%–28.4%) had an additional 18 episodes of CDI recurrence. Baseline demographic, disease, and treatment characteristics of the patients with recurrent CDI are presented in **Table 1** **.**

**Table 1:** Demographic and treatment characteristics of patients with first and later episodes of *Clostridium difficile* infection recurrence.

Characteristic		First recurrence	Second or later recurrence
		(n = 64)	(n = 18)
**Age (years, mean±standard deviation)**		72.9±14.3	64±13.7
**Female gender**		53.1 (40.9–65.3)	58.3 (35.5–81.1)
**Severe CDI** *		28.1 (17.1–39.1)	33.3 (11.5–55.1)
**Treatment** **			
	Metronidazole	36.4 (24.6–48.2)	100.0
	Vancomycin	39.4 (27.4–51.4)	0.0
Both vancomycin and metronidazole		24.2 (13.7–34.7)	0.0

Numbers respresent percent (95% CI) unless otherwise stated.

*No patients who were treated on a strictly outpatient basis were classified as having severe disease.

**Information on treatment of CDI was available only for patients who were treated as inpatients.

**Figure 1:**
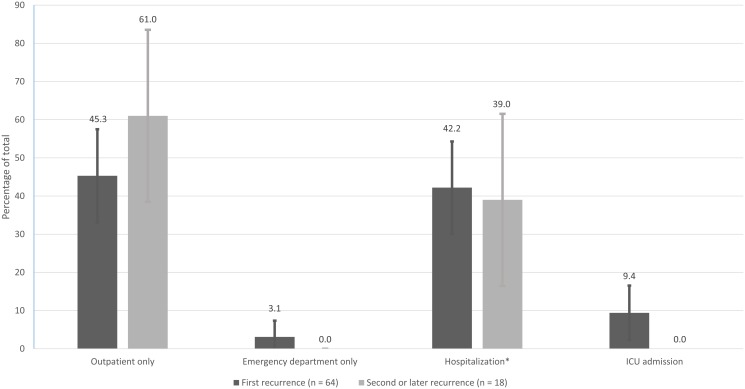
Location of care for patietns with recurrent *Clostridium difficile* infection. *Patients hospitalized without direct ICU admission.

Among patients with recurrent CDI, 29 (45.3%, 95% CI: 33.0%–57.5%) received treatment on a strictly outpatient basis without subsequent re-hospitalization. Two patients (3.1%, 95% CI: 0.0%–7.4%) had an emergency department visit and were not admitted. Thirty-three (51.6%, 95% CI: 39.4%–63.8%) of the initial cohort required hospitalization, of whom 6 (18.2%, 95% CI: 5.0%–31.4%) were directly admitted to an ICU. When using a strict *a priori* definition of CDI-attributable readmission used in this study, 19 (57.6%, 95% CI: 40.7%–74.5%) of readmissions were considered to be due directly to CDI. Characteristics of the type of care obtained by patients with first and later recurrences of CDI are summarized in **Figure 1**. The median time to an initial recurrence was 30 days for the initial positive toxin test, with an interquartile range of 32 days. 30 patients (46.9%, 95% CI: 34.7%–59.1%) of the cohort experienced an initial recurrence within 30 days of the initial positive toxin test, 9 (14.1%, 95% CI: 5.6%–22.6%) of whom were re-hospitalized within 30 days.

**Figure 2:**
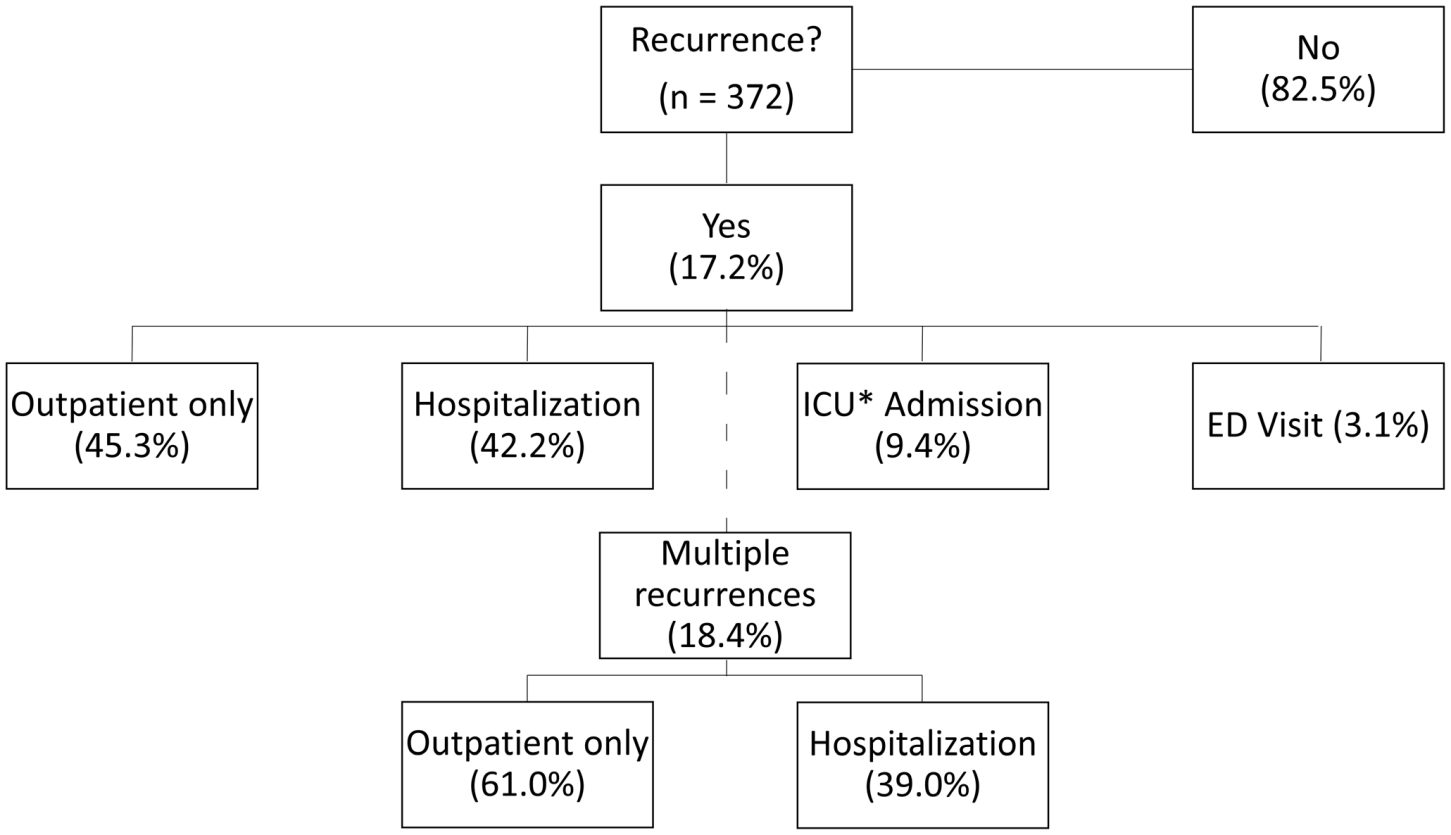
Summary of healthcare reosurces utilized by patients with recurrent *Clostridium difficile* infection. *ICU = Intensive care unit.

For patients with an initial CDI recurrence who required hospitalization, the median (± interquartile range) length of stay was 10±16 days, with a median ICU length of stay of 9±18 days. One of the 64 patients (1.6%, 95% CI: 0.0%–4.7%) died during the hospitalization for recurrent CDI, while an additional two (3.2%, 95% CI: 0.0%–7.5%) patients were discharged to hospice. The length of stay varied when CDI-attributable readmission was assessed (**Table 2**). The median (± interquartile range) length of stay for patients with a readmission attributable to recurrent CDI was 6±6 days. For patients admitted who experienced recurrent CDI >3 days after hospital admission, the length of hospital stay was 22±28 days. Of the 33 patients who were admitted with recurrent CDI, 28 (84.8%, 95% CI: 72.6%–97.1%) were admitted to the initial hospital for medical care and 17 (51.5%, 95% CI: 34.5%–68.6%) required a higher level of care upon discharge. Summary statistics for the three-month follow-up and medical care required for the entire cohort are available in **Figure 2**.

**Table 2:** Length of stay estimates for inpatients with recurrent CDI*.

Parameter	Admission attributable to recurrent CDI only	Recurrent CDI >3 days into hospital admission
**Length of stay (days), median (± IQR** ** **)**	6 (6)	22 (28)

*CDI = Clostridium difficile infection.

**IQR = Interquartile range.

## Discussion

This study was conducted in order to better understand the healthcare resources utilized by patients with recurrent CDI. Our analysis was completed prospectively in a real-world setting outside of the context of a clinical trial including 3-month follow-up data. We found that recurrent CDI leads to a substantial amount of healthcare resource utilization in a number of different settings. These results indicate that patients with recurrent CDI obtain follow-up through a variety of means and indicate a need for more systematic tracking of the resources required to care for this difficult population.

Slightly greater than half of patients with recurrent CDI obtained care in an inpatient setting, while the remainder utilized solely outpatient care. Of these readmissions, half could be attributed to CDI recurrence using strict definition criteria. A prior study found that 21.6% of inpatients diagnosed with CDI will have a subsequent readmission within 180 days [4]. This is larger than the 9.1% overall rate than we found, however, there are two major differences between this study and ours. First, the overall recurrence rate in relation to the initial cohort of the prior study is unknown. Second, and more importantly, the follow-up period was twice the length of ours, potentially increasing the rate of total hospital readmissions [11]. Additionally, our study was able to demonstrate admissions which were attributable directly to CDI using a strict criteria.

We also assessed hospital length of stay for patients with re-hospitalizations due to recurrent CDI. For patients with an admission directly attributable to CDI, the length of stay was similar to previously reported data attributed to primary CDI [4], [5], [12], [13]. Importantly, other studies that have assessed the length of stay burden of recurrent CDI have used statistical methods or total hospitalization rather than direct attribution to determine the economic impact of recurrent CDI [4], [5].

Our study has many limitations. Despite prospective, longitudinal follow-up, we were unable to further qualify the nature of outpatient care that was obtained by patients with recurrent CDI. However, a quantitative assessment demonstrated that roughly half of all patients utilized strictly outpatient care indicating the need for further study of this population. Additionally, our study was based at a single, high-volume tertiary care center located within a major urban center. Therefore the results on the frequency of readmission to the initial study center may not be broadly applicable to all healthcare institutions. Finally, the requirement to have had CDI listed as the primary admission diagnosis to meet our criteria for CDI-attributable readmission may have led to an underestimate of the true rate of readmissions attributable to CDI. In certain cases, the presence of CDI in addition to multiple medical comorbidities or an exacerbation of a chronic condition may have led to the decision to hospitalize, with a comorbidity listed as the primary admission diagnosis.

In conclusion, we have demonstrated that recurrent CDI leads to substantial healthcare resource utilization. For those readmitted with recurrent CDI, the length of stay of recurrent CDI is similar to that attributable to primary CDI. Further studies are needed in order to identify methods to reduce the healthcare burden of recurrent CDI.
